# Upper Extremity Exercise Capacity and Activities of Daily Living in Individuals With Bronchiectasis Versus Healthy Controls

**DOI:** 10.1093/ptj/pzad012

**Published:** 2023-02-01

**Authors:** Aslihan Cakmak, Elif Kocaaga, Hazal Sonbahar-Ulu, Deniz Inal-Ince, Naciye Vardar-Yagli, Ebru Calik-Kutukcu, Melda Saglam, Lutfi Coplu

**Affiliations:** Faculty of Physical Therapy and Rehabilitation, Hacettepe University, Ankara, Turkey; Faculty of Physical Therapy and Rehabilitation, Hacettepe University, Ankara, Turkey; Department of Physiotherapy and Rehabilitation, Faculty of Health Sciences, Akdeniz University, Antalya, Turkey; Faculty of Physical Therapy and Rehabilitation, Hacettepe University, Ankara, Turkey; Faculty of Physical Therapy and Rehabilitation, Hacettepe University, Ankara, Turkey; Faculty of Physical Therapy and Rehabilitation, Hacettepe University, Ankara, Turkey; Faculty of Physical Therapy and Rehabilitation, Hacettepe University, Ankara, Turkey; Department of Chest Diseases, Faculty of Medicine, Hacettepe University, Ankara, Turkey

**Keywords:** Bronchiectasis, Daily Living Activities, Exercise Capacity

## Abstract

**Objective:**

The purpose of this study was to compare the upper extremity exercise capacity and activities of daily living (ADL) in individuals with bronchiectasis and controls.

**Methods:**

Twenty-four individuals with bronchiectasis and 24 healthy controls were assessed for upper extremity exercise capacity (6-minute pegboard and ring test [6PBRT]) and ADL (Glittre ADL test). Energy expenditure was measured using a wearable metabolic monitor during the Glittre ADL test.

**Results:**

The mean [SD] 6PBRT score of individuals with bronchiectasis was significantly lower than the mean score of controls (196.50 [51.75] vs 243.00 [29.76] number of rings). The Glittre ADL test duration was significantly higher in individuals with bronchiectasis compared with controls (3.54 [1.53] vs 2.36 [0.18] minutes), despite similar energy expenditure during the Glittre ADL test between the groups (17.67 [5.28] kcal in individuals with bronchiectasis vs 18.13 [5.71] kcal in controls). The 6PBRT score and the Glittre ADL test duration were negatively correlated in individuals with bronchiectasis (*r* = −0.694).

**Conclusion:**

The individuals with bronchiectasis had reduced upper extremity exercise capacity compared with healthy controls. Energy expenditure during ADL was similar between individuals with bronchiectasis and healthy controls, despite lower ADL performance in individuals with bronchiectasis. The upper extremity exercise capacity and ADL are related in individuals with bronchiectasis. Given this relationship, inclusion of upper extremity exercise training in pulmonary rehabilitation programs should be considered.

**Impact:**

Considering the impairment of upper extremity exercise capacity and ADL in individuals with bronchiectasis highlights the need to tailor preventive strategies and preclude further unfavorable effects.

**Lay Summary:**

Bronchiectasis may reduce exercise capacity in your arms and reduce your ability to perform daily living activities. Physical therapists can evaluate your condition and create rehabilitation programs to help manage these impairments.

## Introduction

Bronchiectasis is similar to chronic obstructive lung disease (COPD) in clinical manifestations, absence of reversibility, and chronic inflammation.^1^ Exercise tolerance, physical activity level, and respiratory and peripheral muscle strength of individuals with bronchiectasis are lower than in healthy controls.^2^

Exercise intolerance occurs not only in performing lower extremity tasks but also manifests itself during arm activities of daily life in COPD.^3^ Respiratory muscle recruitment pattern changes as greater respiratory load shifts from the inspiratory muscles of the rib cage to the diaphragm and expiratory muscles during unsupported arm exercise.^4^ Unsupported arm exercise is associated with a change in breathing pattern and precipitates dyspnea in individuals with chronic airflow obstruction.^4^

Upper extremity exercise simulates daily living arm activities.^5^ Because accessory respiratory muscles during upper extremity exercise cannot contribute to respiration, the respiratory load of the mechanically disadvantaged diaphragm increases, resulting in thoracoabdominal synchrony and severe dyspnea.^3^^,^^6^ Because the muscles moving the upper extremity and stabilizing the trunk are attached to the rib cage, they increase the resistance of the chest wall and limit the ability to increase tidal volume during upper extremity activities.^3^^,^^6^ These disturbances in ventilatory mechanics in COPD cause upper extremity exercise to be terminated at low workloads compared with healthy controls.^3^^,^^7^

Six-minute pegboard and ring test (6PBRT) performance is related to activities of daily living (ADL) in individuals with airflow obstruction such as COPD and asthma.^8^^,^^9^ The 6PBRT is used to evaluate unsupported upper extremity exercise capacity, and is a predictive test to maintain and improve upper extremity ADL during pulmonary rehabilitation.^9^

Adults with bronchiectasis with reduced exercise capacity are recommended to exercise regularly and participate in a pulmonary rehabilitation program, taking into account their symptoms, physical capacities, and disease characteristics.^10^ There is no evidence about the extent to which the upper extremity is affected in individuals with bronchiectasis in the framework of monitoring and management of clinical status in the context of pulmonary rehabilitation.

Glittre ADL tests are used to determine ADL in COPD. The Glittre ADL test encompasses activities necessary for daily living and commonly used activities that are known to be difficult for individuals with COPD. Although there are studies in which ADL were determined using the Glittre ADL test in COPD,^5^^,^^9^ there is only 1 study conducted in individuals with bronchiectasis.^11^ The Glittre ADL test produces cardiorespiratory responses comparable to the 6-Minute Walk Test.^11^

The upper extremity exercise capacity and ADL of individuals with bronchiectasis may differ from healthy individuals. There is limited information comparing upper extremity exercise capacity and ADL between individuals with bronchiectasis and healthy controls. Although the Glittre ADL test has been shown to be a discriminative test in individuals with bronchiectasis compared with healthy controls,^10^ no study has used the 6PBRT to determine the upper extremity exercise capacity in bronchiectasis. This study’s primary objective was to compare the upper extremity exercise capacity and ADL of individuals with bronchiectasis and healthy controls and to show the discriminative properties of the 6PBRT in bronchiectasis. The secondary objectives were to compare energy expenditure during the Glittre ADL test of individuals with bronchiectasis and healthy controls, and to investigate the relationship between upper extremity exercise capacity and ADL in individuals with bronchiectasis.

## Methods

This cross-sectional study was carried out at Hacettepe University, Faculty of Physical Therapy and Rehabilitation, Cardiopulmonary Rehabilitation Unit, between January 2020 and March 2022.

Hacettepe University Non-Interventional Clinical Research Ethical Committee approved the study, with the approval number of GO 20/55 and approval date of January 21, 2020. Signed informed consent was obtained from all participants.

The inclusion criteria were as follows: being diagnosed with bronchiectasis at Hacettepe University, Faculty of Medicine, Department of Chest Diseases and routinely referred to Hacettepe University, Faculty of Physical Therapy and Rehabilitation, Cardiopulmonary Rehabilitation Unit for a physical therapy and rehabilitation program; being clinically stable; being 18 years old or older; and being able and willing to complete the informed consent process. The diagnosis of bronchiectasis was established based on high-resolution computed tomography.^12^

Exclusion criteria were: individuals with bronchiectasis whose clinical condition was unstable; who had severe neuromuscular, musculoskeletal, and rheumatological problems; were unable to cooperate; or were unwilling to participate in the study. The healthy controls consisted of individuals without any known disease. Healthy individuals who did not volunteer to participate in the study were excluded. The individuals with bronchiectasis had not attended a rehabilitation program recently or at the time of evaluations.

### Assessments

The physical characteristics (height and weight) of participants were recorded. A pulmonary function test was performed using a spirometer (Spirodoc; Medical International Research, Rome, Italy). Forced vital capacity (FVC), forced expiratory volume in 1 second (FEV_1_), the ratio FEV_1_/FVC, peak expiratory flow (PEF), and forced mid-expiratory flow rate (FEF_25–75%_) were recorded.^13^ European Respiratory Society Global Lung Function Initiative reference values were used for spirometric variables.^14^ The severity of lung function impairment was classified based on the FEV_1_% predicted (>70 mild, 60–69 moderate, 50–59 moderately severe, 35–49 severe, <35 very severe).^15^

### Upper Extremity Exercise Capacity

Upper extremity exercise capacity was evaluated using 6PBRT. The test is performed in a sitting position in front of a pegboard. Participants are requested to move as many rings as possible from the 2 lower holes to the 2 upper holes, using both hands simultaneously, for 6 minutes. Standardized encouragement is given every minute during the test.^16^ The number of rings carried during the 6 minutes constitutes the final score.^5^ A lower 6PBRT score represents a smaller number of rings moved during the 6PBRT. The 6PBRT was performed twice with at least 30 minutes of rest between tests.^5^ The test parameters with the best test score were used for analyses. The percentage of predicted 6PBRT score was calculated based on reference values.^17^ Before and after the test, heart rate (HR) and oxygen saturation (SpO_2_) were recorded using a portable pulse oximeter (Nonin Palmsat 2550A; Nonin Medical Inc, Plymouth, MN, USA). Dyspnea perception, upper extremity fatigue, and general fatigue using the modified Borg scale were recorded.^5^^,^^9^^,^^16^

### Activities of Daily Living

The Glittre ADL test covers the activities necessary for daily living and commonly used activities that are known to be difficult for individuals with COPD.^18^ The individual starts the test in a sitting position, walks up and down over 2 steps, and walks to the shelf individually adjusted according to the shoulder and waist height.^18^ Three 1-kg objects on the upper shelf are moved to the lower shelf, then to the floor, back to the lower shelf, and finally to the upper shelf. The individual then turns around, walks back to the chair, sits down, gets up quickly, and starts the next round.^18^ The test includes 5 rounds on a 10-m distance, and individuals are asked to complete these rounds as quickly as possible.^18^ Females carry a 2.5-kg backpack during the test, and males carry a 5-kg backpack.^18^ Each step of the ladder is 17 cm high and 27 cm wide. The test completion time is recorded in minutes.^18^ Before and after the test, HR and SpO_2_ values were recorded using a portable pulse oximeter (Nonin Palmsat 2550A). Before and after the test, dyspnea perception and leg and general fatigue using the modified Borg scale were recorded.^11^ The Glittre ADL test was performed twice, with at least 30 minutes of rest between tests.^11^ The test parameters with the best test score were used for analyses. During the Glittre ADL test, energy expenditure was measured using a metabolic holter device (SenseWear Armband Model MF-SW; BodyMedia, Pittsburgh, PN, USA).^19^ The SenseWear Armband is a useful tool for determining energy expenditure at low intensities, even though it is not as precise in estimating energy expenditure as indirect calorimetry.^20^

### Statistical Analysis

The sample size and post hoc power analysis were calculated using the G*Power statistical program 3.1.9.6 (Heinrich Heine University, Düsseldorf, Germany). Power analysis with a 2-tailed type 1 error of 0.05 and a power of 90% showed that to detect a difference in Glittre ADL test duration between individuals with bronchiectasis and healthy controls required 24 participants per group.^21^ The Statistical Package for Social Sciences (SPSS) version 25.0 statistical analysis program (IBM Corp, Armonk, NY, USA) was used to analyze the data. Descriptive statistics were presented. The analyses were performed considering the conformity of the data to the normal distribution. Parametric data were analyzed using Student *t* test, nonparametric data were compared using the Mann-Whitney *U* test. Pearson or Spearman correlation coefficients depending on normality were used to determine the relationships between parameters. The statistical significance level^22^ for all tests was determined as *P* < .05. Post hoc power, calculated using the Glittre ADL test duration, revealed Cohen *d* as 1.08 and 1 − β as 95.66%.

## Results

Twenty-four individuals with bronchiectasis (15 females, 9 males; mean [SD] age = 34 [18] years) and 24 healthy controls (14 females, 10 males; mean age = 29 [8] years) were included. Two of the individuals with bronchiectasis (8.3%) were ex-smokers, and 22 of them (91.7%) were nonsmokers, whereas 7 of the controls (29.2%) were active smokers. One of the controls (4.2%) was an ex-smoker, and 16 of the controls (66.7%) were nonsmokers. The individuals with bronchiectasis had a lower smoking history in terms of pack-years than healthy controls (mean [SD] = 0.88 [3.70] vs 3.00 [7.17], *P* = .041).

The underlying etiologies of bronchiectasis of the included individuals were classified as immunodeficiency in 8 subjects (n = 1 autoimmune polyendocrinopathy-candidiasis-ectodermal dystrophy, n = 3 hypogammaglobulinemia, n = 1 lymphocyte function-related antigen-1 deficiency, and n = 3 unspecified immunodeficiency); mucociliary insufficiency in 6 individuals (n = 4 primary ciliary dyskinesia and n = 2 Kartagener syndrome); and postinfectious bronchiectasis in 10 individuals (n = 1 adulthood bacterial pneumonia, n = 1 measles, n = 1 adenovirus infection, and n = 7 childhood bacterial pneumonia). Considering the lobe distribution of bronchiectasis using high-resolution computed tomography results, the right upper lobe was involved in 5 (20.8%), the right middle lobe was involved in 13 (54.2%), the right lower lobe was involved in 18 (75.0%), the left upper lobe was involved in 13 (54.2%), and the left lower lobe was involved in 16 (66.7%). Of the individuals with bronchiectasis, there was single-lobe involvement in 3 (12.5%), 2-lobe involvement in 11 (45.8%), 3-lobe involvement in 3 (12.5%), 4-lobe involvement in 4 (16.7%), and 5-lobe involvement in 3 (12.5%).

The participants’ physical characteristics and pulmonary function parameters are presented in Table 1. The predicted percentages of FVC, FEV_1_, FEV_1_/FVC, PEF, and FEF_25–75%_ of the individuals with bronchiectasis were significantly lower than the values of healthy controls (*P* < .05, Tab. 1). More than half of the individuals with bronchiectasis (66.7%) had bronchiectasis with mild lung function impairment, whereas 1 (4.2%), 3 (12.5%), 2 (8.3%), and 2 (8.3%) had moderate, moderately severe, severe, and very severe lung function impairment, respectively.

**Table 1:** Characteristics of Participants*^a^*

Parameters	Individuals With Bronchiectasis (n = 24)	Controls (n = 24)	*P*
	Mean [SD]		
Age, y	34 [18]	29 [8]	.749*^b^*
Sex F/M, n (%)	15/9 (62.5/37.5)	14/10 (58.3/41.7)	.768*^c^*
Height, m	1.67 [0.09]	1.68 [0.09]	.701*^d^*
Weight, kg	63.21 [14.95]	63.96 [12.10]	.849*^d^*
Body mass index, kg/m^2^	22.82 [5.51]	22.88 [4.75]	.773*^b^*
FVC, L	2.95 [1.02]	3.81 [0.93]	.004*^e^*
FVC, %	75.08 [17.32]	92.63 [12.49]	<.001*^b^**^,^**^e^*
FEV_1_, L	2.48 [1.11]	3.32 [0.82]	.004*^e^*
FEV_1_, %	71.58 [22.19]	94.13 [11.50]	<.001*^b^**^,^**^e^*
FEV_1_/FVC	80.04 [10.34]	87.73 [6.46]	.003*^e^*
PEF, L/min	5.46 [2.48]	6.80 [2.19]	.053*^d^*
PEF, %	68.38 [22.63]	84.21 [20.50]	.015*^e^*
FEF_25–75%_, L	2.40 [1.40]	3.68 [1.21]	<.001*^b^**^,^**^e^*
FEF_25–75%_, %	56.96 [28.38]	84.13 [22.46]	.001*^e^*

^a^ F, female; FEF_25–75%_ = forced expiratory flow at 25–75% of the vital capacity; FEV_1_ = forced expiratory volume in 1 second; FVC = forced vital capacity; M = male; PEF = peak expiratory flow.

^b^ Mann-Whitney *U* test

^c^ Chi-square test.

*^d^* Student *t* test.

^e^ *P* < .05

The HR, SpO_2_, systolic and diastolic blood pressure, dyspnea perception, upper extremity fatigue, and general fatigue before and after 6PBRT are shown in Table 2. All parameters except SpO_2_ were significantly increased after 6PBRT in both individuals with bronchiectasis and healthy controls (*P* < .01, Tab. 2).

**Table 2:** The Comparison of Pre- and Post-6-Minute Pegboard and Ring Test and Glittre Activities of Daily Living Test Values of Individuals with Bronchiectasis and Healthy Controls*^a^*

Parameters	Individuals With Bronchiectasis (n = 24)			Controls (n = 24)		
	Pretest	Posttest	*P*	Pretest	Posttest	*P*
	Median (IQR) or Mean [SD]			Median (IQR) or Mean [SD]		
*6PBRT* *^b^*						
Heart rate, bpm	89.33 [13.81]	102.50 [16.06]	<.001*^c^**^,^**^d^*	83.96 [10.14]	99.75 [12.93]	<.001*^c^**^,^**^d^*
SpO_2_, %	94.0 (12.0)	95 (10.0)	.095*^e^*	96.0 (16.0)	96.5 (17.0)	.069*^e^*
SBP, mmHg	108.0 (50.0)	120.0 (48.0)	<.001*^c^**^,^**^e^*	105.0 (52.0)	114.0 (50.0)	<.001*^c^**^,^**^e^*
DBP, mmHg	67.0 (40.0)	71.0 (32.0)	.003*^c^**^,^**^e^*	69.0 (46.0)	70.0 (41.0)	.004*^c^**^,^**^e^*
Dyspnea, 0–10	0.0 (7.0)	2.0 (7.0)	.001*^c^**^,^**^e^*	0.0 (0.0)	0.0 (3.0)	.004*^c^**^,^**^e^*
General fatigue, 0–10	0.0 (8.0)	2.0 (8.0)	.002*^c^**^,^**^e^*	0.0 (1.0)	0.5 (5.0)	.003*^c^**^,^**^e^*
Arm fatigue, 0–10	0.0 (3.0)	5.0 (9.0)	<.001*^c^**^,^**^e^*	0.0 (1.0)	3.5 (8.0)	<.001*^c^**^,^**^e^*
*Glittre ADL test* *^b^*						
Heart rate, bpm	92.42 [11.24]	130.79 [30.54]	<.001*^c^**^,^**^d^*	84.75 [13.53]	139.13 [19.26]	<.001*^c^**^,^**^d^*
SpO_2_, %	95.0 (10.0)	93.5 (24.0)	.001*^c^**^,^**^e^*	97.0 (5.0)	96.0 (6.0)	.002*^c^**^,^**^e^*
SBP, mmHg	108.5 [14.65]	127.79 [12.23]	<.001*^c^**^,^**^d^*	103.42 [11.37]	123.25 [14.46]	<.001*^c^**^,^**^d^*
DBP, mmHg	67.0 (37.0)	70.0 (38.0)	.003*^c^**^,^**^e^*	63.0 (24.0)	70.0 (44.0)	.022*^c^**^,^**^e^*
Dyspnea, 0–10	0.0 (3.0)	3.0 (9.0)	<.001*^c^**^,^**^e^*	0.0 (1.0)	2.0 (4.0)	<.001*^c^**^,^**^e^*
Leg fatigue, 0–10	0.0 (3.0)	1.5 (6.0)	.001*^c^**^,^**^e^*	0.0 (1.0)	1.0 (4.0)	<.001*^c^**^,^**^e^*
General fatigue, 0–10	0.0 (5.0)	3.0 (7.0)	<.001*^c^**^,^**^e^*	0.0 (1.0)	1.0 (3.0)	.001*^c^**^,^**^e^*

^a^ bpm, beats per minute; DBP = diastolic blood pressure; Glittre ADL test = Glittre activities of daily living test; IQR = interquartile range; SBP = systolic blood pressure; SpO_2_ = saturation of oxygen; 6PBRT = 6-minute pegboard and ring test.

^b^ Data are presented as mean [SD] or median (IQR).

^c^ *P* < .05.

*^d^* Paired sample *t* test.

^e^ Wilcoxon signed rank test.

The comparisons of post- and pretest differences in the 6PBRT and the Glittre ADL test between individuals with bronchiectasis and healthy controls are shown in Table 3. The mean 6PBRT score and the predicted percentage of 6PBRT score of individuals with bronchiectasis was significantly lower than those of healthy controls (*P* < .001, Tab. 3). There were no statistically significant differences between the 2 groups in post- and pre-6PBRT in HR, SpO_2_, systolic and diastolic blood pressure, dyspnea perception, and general fatigue. The 6PBRT upper extremity fatigue difference of individuals with bronchiectasis was significantly higher than that of controls (*P* < .05, Tab. 3).

**Table 3:** The Comparison of 6-Minute Pegboard and Ring Test and Glittre Activities of Daily Living Test Between Individuals with Bronchiectasis and Healthy Controls*^a^*

Parameters	Individuals with Bronchiectasis (n = 24)	Controls (n = 24)	*P*
	Median (IQR) or Mean [SD]		
*6PBRT* *^b^*			
ΔHeart rate, bpm	13.17 [7.81]	15.79 [9.53]	.302*^c^*
ΔSpO_2_, %	0.0 (5.0)	0.0 (5.0)	.856*^d^*
ΔSBP, mmHg	10.0 (40.0)	8.0 (35.0)	.532*^d^*
ΔDBP, mmHg	5.0 (32.0)	5.5 (33.0)	.934*^d^*
ΔDyspnea, 0–10	0.5 (3.5)	0.0 (3.0)	.174*^d^*
ΔGeneral fatigue, 0–10	0.5 (4.5)	0.0 (5.0)	.431*^d^*
ΔArm fatigue, 0–10	5.0 (9.0)	3.5 (8.0)	.019*^d,e^*
6PBRT score, number of rings	205 (196)	245 (95)	<.001*^d,e^*
6PBRT score % predicted, %	76.2 (62.4)	88.1 (32.4)	.001*^d,e^*
*Glittre ADL test* *^b^*			
ΔHeart rate, bpm	38.38 [25.45]	54.38 [13.13]	.009*^c,e^*
ΔSpO_2_, %	−3.0 (22.0)	−1.0 (6.0)	.043*^d,e^*
ΔSBP, mmHg	19.29 [13.06]	19.83 [10.47]	.875*^c^*
ΔDBP, mmHg	5.88 [8.19]	4.42 [8.09]	.538*^c^*
ΔDyspnea, 0–10	3.0 (7.0)	2.0 (4.0)	.002*^d,e^*
ΔLeg fatigue, 0–10	1.5 (5.0)	1.0 (4.0)	.750*^d^*
ΔGeneral fatigue, 0–10	3.0 (5.0)	1.0 (3.0)	<.001*^d,e^*
Test duration, min	3.18 (5.93)	2.33 (1.01)	<.001^d,e^
Energy expenditure, kcal	17.67 [5.28]	18.13 [5.71]	.774*^c^*

^a^ bpm = beats per minute; DBP = diastolic blood pressure; Glittre ADL test = Glittre activities of daily living test, IQR = interquartile range; SBP = systolic blood pressure; SpO_2_ = saturation of oxygen; Δ = difference; 6PBRT = 6-minute pegboard and ring test.

^b^ Data are presented as mean [SD] or median (IQR).

^c^ Student *t* test.

^d^ Mann-Whitney *U* test.

^e^ *P* < .05.

The comparisons of pre- and post-Glittre ADL test values and the change in Glittre ADL test values of the participants are given in Table 2. The HR, systolic and diastolic blood pressure, dyspnea perception, leg fatigue, and general fatigue values after the Glittre ADL test were significantly higher than pretest values in both groups (*P* < .05, Tab. 2). The SpO_2_ was significantly decreased with the Glittre ADL test in both groups (*P* < 0.01, Tab. 2).

The Glittre ADL test duration was significantly greater in individuals with bronchiectasis compared with controls (*P* < .001, Tab. 3), even though the energy expenditure during the Glittre ADL test was similar between the groups (*P* = .774, Tab. 3). The Glittre ADL test HR difference was significantly lower in individuals with bronchiectasis compared with healthy controls (*P* < .01, Tab. 3). The post- and pre-Glittre ADL test differences in SpO_2_ were significantly lower in individuals with bronchiectasis (*P* < .05, Tab. 3). The post- and pre-Glittre ADL test differences in systolic and diastolic blood pressure and leg fatigue were similar between the 2 groups (*P* > .05, Tab. 2). The post- and pre-Glittre ADL test differences in terms of dyspnea perception and general fatigue were significantly higher in individuals with bronchiectasis than in healthy control (*P* < .01, Tab. 3).

**Figure 1:**
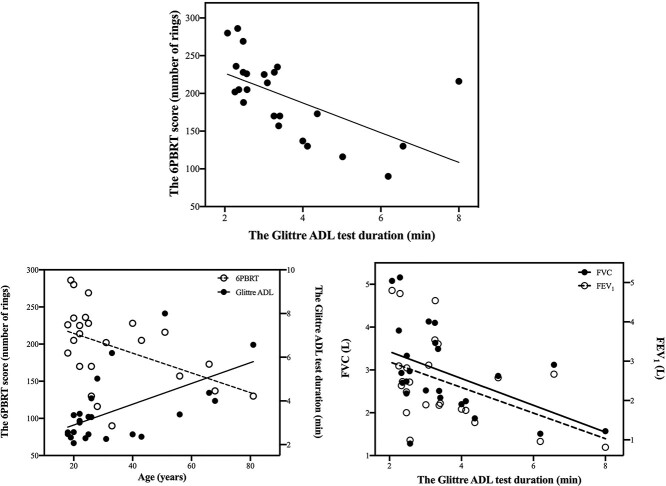
The correlations between the 6PBRT score and Glittre ADL test duration, the 6PBRT score and Glittre ADL test duration with age, and FEV_1_ and FVC with the Glittre ADL test duration in individuals with bronchiectasis. ADL = activities of daily living; FEV_1_ = forced expiratory volume in 1 second; FVC = forced vital capacity; 6PBRT = 6-minute pegboard and ring test.

There was a negative correlation between the 6PBRT score and Glittre ADL test duration in individuals with bronchiectasis (*r* = −0.694, *P* < .001) (Fig. 1). In contrast, no significant correlation was present between the 6PBRT score and Glittre ADL test duration in healthy controls (*r* = −0.216, *P* = .311). There was a negative correlation between FEV_1_ and FVC with the Glittre ADL test duration in individuals with bronchiectasis (*r* = −0.469, *P* = .021; *r* = −0.527, *P* = .008, respectively), whereas there were no significant relationships between pulmonary function test parameters with 6PBRT and the Glittre ADL test in controls (*P* > .05). Age was negatively correlated with 6PBRT score (*r* = −0.540, *P* = .006) and positively correlated with Glittre ADL test duration in individuals with bronchiectasis (*r* = 0.515, *P* = .010) (Fig. 1).

## Discussion

To the best of our knowledge, this is the first study presenting the 6PBRT responses and showing the discriminative property of the 6PBRT in individuals with bronchiectasis when comparing its outcomes with those of healthy controls. The individuals with bronchiectasis had reduced upper extremity exercise capacity than their age-matched healthy controls. Even though energy expenditure during the Glittre ADL test was similar between the 2 groups, the individuals with bronchiectasis had impaired Glittre ADL test performance compared with healthy controls.

There are no studies that determine upper extremity exercise capacity using 6PBRT in bronchiectasis. The 6PBRT score of individuals with bronchiectasis was lower than controls (mean [SD] = 196.5 [51.7] vs 243.0 [29.7]). The arm fatigue of individuals with bronchiectasis after 6PBRT was significantly higher than that of controls. Patients with bronchiectasis have been shown to have lower strength in biceps brachii, shoulder flexors, and shoulder abductors compared with healthy counterparts.^2^^,^^23^ Zhan et al^5^ showed that dyspnea and arm fatigue response to 6PBRT were higher in individuals with COPD than in healthy controls. Arm muscle dysfunction might explain why individuals with bronchiectasis have lower 6PBRT scores and higher arm fatigue compared with controls. Furthermore, the respiratory load shifts from the inspiratory muscles of the ribcage to the diaphragm and expiratory muscles during unsupported upper extremity exercise in individuals with chronic airflow limitation.^4^ These altered breathing mechanics might be the mechanism behind the impaired upper extremity exercise capacity in individuals with bronchiectasis compared with controls. Lima et al^24^ reported that younger individuals had better 6PBRT performance compared with older individuals. The relatively younger individuals with bronchiectasis with mild airflow obstruction included in our study might be a reason for the similar dyspnea perception between groups.

The HR, blood pressure, dyspnea perception, and arm and general fatigue increased after 6PBRT in both groups compared with the pretest values in 6PBRT. However, no significant change was observed in SpO_2_ in both groups. To our knowledge, there is no study investigating the physiological responses to 6PBRT in bronchiectasis. Physiological responses were evaluated before and after 6PBRT in COPD, but initial measurements were not reported.^5^ A study of individuals with asthma showed increased HR, blood pressure, dyspnea, and fatigue after 6PBRT compared with baseline values.^8^ The current findings in bronchiectasis were similar to these previous results. However, we did not observe a significant difference in SpO_2_ after the test in both groups. The fact that 6PBRT did not cause desaturation may suggest that it is safe to use in clinical practice.

We found that the Glittre ADL test performance of individuals with bronchiectasis was lower than that of healthy controls, consistent with the literature.^11^ Individuals with bronchiectasis have lower peripheral muscle strength, exercise capacity, and physical activity levels than healthy controls.^2^^,^^23^ Peripheral muscle strength and aerobic capacity are related in individuals with bronchiectasis,^23^ and Glittre ADL test duration is related to functional exercise capacity.^11^ The reduction in peripheral muscle strength, exercise capacity, and physical activity may be reasons for the decreased ADL. Our previous work showed similar Glittre ADL test durations in individuals with bronchiectasis and healthy controls. This probably was the result of the relatively small sample size in the aforementioned study (12 individuals with bronchiectasis vs 10 controls)^25^ compared with the current study (24 individuals with bronchiectasis vs 24 controls).

Even though the individuals with bronchiectasis had impaired Glittre ADL test performance compared with healthy controls, energy expenditure during the Glittre ADL test was similar between groups. In our previous study, we found that the energy expenditure was similar between groups although the average duration of moderate and vigorous activity of individuals with bronchiectasis was less than the healthy controls.^2^ Individuals with chronic lung disease were shown to have a disproportionate increase in ventilation for a given mechanical workload and oxygen cost during exercise.^26^ The low performance with a preserved energy expenditure during the Glittre ADL test may be attributable to the oxygen cost of increased ventilation during exercise.^26^ We interpreted this finding previously as the individuals with bronchiectasis may have spent more energy at a given workload than healthy controls.^2^ The current findings seem to support this conclusion.

The change in HR with the Glittre ADL test was lower in individuals with bronchiectasis compared with the controls. Contrary to the present study findings, it has been shown that the HR response to the Glittre ADL test is similar in individuals with bronchiectasis and healthy controls.^11^ However, Hena et al^11^ included older participants, both those with bronchiectasis and controls (mean [SD] age = 50.8 [11.55] vs 50.7 3 [11.47] years) compared with the participants of our study (33.50 [18.02] vs 28.92 [7.76] years), and they reported a statistically significant difference between groups in terms of weight and body mass index. On the other hand, in the aforementioned study,^11^ SpO_2_ decreased in the group of patients with bronchiectasis more than the controls in response to the Glittre ADL test, similar to our findings. A decrease in SpO_2_ induced by exercise is expected in chronic lung diseases.^11^ In the present study, the dyspnea and fatigue perception in individuals with bronchiectasis increased more than in the control group. Decreased SpO_2_, increased dyspnea, and fatigue perception with the Glittre ADL test could result from deconditioning, muscle dysfunction, impaired functional exercise capacity, and dynamic hyperinflation with increased work of breathing.^2^^,^^11^^,^^27^^,^^28^ Hena et al^11^ reported cardiorespiratory responses to the Glittre ADL test in individuals with bronchiectasis and healthy controls. However, they did not compare the pre- and posttest cardiorespiratory responses to the Glittre ADL test in individuals with bronchiectasis.^11^ The present study showed increases in HR, blood pressure, dyspnea, and both quadriceps and general fatigue, and a decrease in SpO_2_ measured after the Glittre ADL test compared with the baseline values in both groups. At the same time, there was a significant decrease in SpO_2_ in both groups. Similar to our findings, a study with individuals with COPD reported a significant increase in HR and decrease in SpO_2_ after the Glittre ADL test compared with baseline values.^29^

The 6PBRT score and upper extremity ADL assessed using a physical activity questionnaire and an accelerometer were related in patients with COPD.^9^ Calik-Kutukcu et al^8^ determined that better 6PBRT scores are related to lower dyspnea scores during unsupported arm activities and self-care ADL and decreased arm activity limitation in patients with asthma. The present study is the first to show a relationship between the 6PBRT score and the Glittre ADL test performance in bronchiectasis.

Individuals with COPD with reduced pulmonary functions have longer Glittre ADL test completion times and dynamic hyperinflation, and premature depletion of inspiratory and expiratory reserves limits the Glittre ADL test performance.^18^^,^^30^^,^^31^ We observed a negative relationship between FEV_1_ and the Glittre ADL test duration. Other than these, age was related to the Glittre ADL test and the 6PBRT score in individuals with bronchiectasis. Age is an independent determinant of the unsupported upper extremity exercise capacity assessed using the 6PBRT in older adults with COPD.^32^ These findings reinforce the necessity to assess upper extremity exercise capacity and ADL even in young adults with bronchiectasis. The 6PBRT can be used to predict ADL performance and provides a useful, practical, and discriminative upper extremity exercise test for individuals with bronchiectasis. The assessment of upper extremity exercise capacity using the 6PBRT in individuals with bronchiectasis can allow early detection of upper extremity functional capacity impairment and provide an important viewpoint in pulmonary rehabilitation in the framework of monitoring and management of the disease.

### Limitations

This study was limited by the absence of the participants’ upper and lower extremity muscle strength and physical activity level assessments. Muscle strength assessment may explain the decreased arm exercise capacity and ADL in individuals with bronchiectasis. Additionally, the majority of individuals with bronchiectasis participating in our study had mild lung function impairment. This should be taken into account in terms of the generalizability of the findings. On the other hand, it reveals that even individuals with bronchiectasis with mild lung function impairment have impaired upper extremity exercise capacity and ADL performance.

There is a need for future studies to determine altered breathing mechanics during unsupported upper extremity activities in individuals with bronchiectasis. This study further creates a basis to investigate the effects of arm exercise training on ADL performance in individuals with bronchiectasis, considering the relationship between arm exercise capacity and ADL performance and the impairment of these in individuals with bronchiectasis.

## Conclusions

In conclusion, individuals with bronchiectasis have reduced upper extremity exercise capacity and impaired daily living performance compared with healthy controls. Energy expenditure during daily living activity appears to be similar in individuals with bronchiectasis and controls, despite lower daily living performance in bronchiectasis. The upper extremity exercise capacity and ADL are related in individuals with bronchiectasis. The 6PBRT could be used as an objective and feasible upper extremity test to predict ADL performance and to determine the need for arm exercise training in individuals with bronchiectasis. Given impaired arm exercise capacity and ADL in bronchiectasis, inclusion of upper extremity exercise training in pulmonary rehabilitation programs should be considered.

## Data Availability

The data that support the findings of this study are available from the corresponding author upon reasonable request.

## Clinical Trial Registration

This study was registered in clinicaltrials.gov (NCT05084547).

## References

[1] Athanazio R . Airway disease: similarities and differences between asthma, COPD and bronchiectasis. Clinics (Sao Paulo). 2012;67:1335–1343. 23184213 10.6061/clinics/2012(11)19 PMC3488995

[2] Cakmak A, Inal-Ince D, Sonbahar-Ulu H, et al. Physical activity of patients with bronchiectasis compared with healthy counterparts: a cross-sectional study. Heart Lung. 2020;49:99–104. 31530430 10.1016/j.hrtlng.2019.09.004

[3] Kathiresan G, Jeyaraman SK, Jaganathan J. Effect of upper extremity exercise in people with COPD. J Thorac Dis. 2010;2:223–236. 22263051 10.3978/j.issn.2072-1439.2010.11.4 PMC3256474

[4] Criner GJ, Celli BR, with the technical assistance of John Rassulo. Effect of unsupported arm exercise on ventilatory muscle recruitment in patients with severe chronic airflow obstruction. Am Rev Respir Dis. 1988;138:856–861. 3202459 10.1164/ajrccm/138.4.856

[5] Zhan S, Cerny FJ, Gibbons WJ, Mador MJ, Wu YW. Development of an unsupported arm exercise test in patients with chronic obstructive pulmonary disease. J Cardiopulm Rehabil. 2006;26:180–187 discussion 188–190. 16738459 10.1097/00008483-200605000-00013

[6] Miranda EF, Malaguti C, Corso SD. Peripheral muscle dysfunction in COPD: lower limbs versus upper limbs. J Bras Pneumol. 2011;37:380–388. 21755195 10.1590/s1806-37132011000300016

[7] McKeough ZJ, Alison JA, Bye PT. Arm exercise capacity and dyspnea ratings in subjects with chronic obstructive pulmonary disease. J Cardiopulm Rehabil. 2003;23:218–225. 12782907 10.1097/00008483-200305000-00010

[8] Calik-Kutukcu E, Tekerlek H, Bozdemir-Ozel C, et al. Validity and reliability of 6-minute pegboard and ring test in patients with asthma. J Asthma. 2022;59:1387–1395. 33985406 10.1080/02770903.2021.1930040

[9] Takeda K, Kawasaki Y, Yoshida K, et al. The 6-minute pegboard and ring test is correlated with upper extremity activity of daily living in chronic obstructive pulmonary disease. Int J Chronic Obstr. 2013;8:347–351. 10.2147/COPD.S45081 PMC3726300 23901268

[10] Polverino E, Goeminne PC, McDonnell MJ, et al. European Respiratory Society guidelines for the management of adult bronchiectasis. Eur Respir J. 2017;50:1700629. 10.1183/13993003.00629-2017 28889110

[11] Hena R, Alaparthi GK, Krishnan KS, Anand R, Acharya V, Acharya P. Cardiorespiratory responses to glittre ADL test in bronchiectasis: a cross-sectional study. Can Respir J. 2018;2018:7470387. 30651896 10.1155/2018/7470387 PMC6311838

[12] Drain M, Elborn JS. Assessment and investigation of adults with bronchiectasis. Eur Respir Monogr. 2011;52:32–43.

[13] Graham BL, Steenbruggen I, Miller MR, et al. Standardization of spirometry 2019 update. An official American Thoracic Society and European Respiratory Society technical statement. Am J Respir Crit Care Med. 2019;200:e70–e88. 31613151 10.1164/rccm.201908-1590ST PMC6794117

[14] Quanjer PH, Stanojevic S, Cole TJ, et al. Multi-ethnic reference values for spirometry for the 3-95-yr age range: the global lung function 2012 equations. Eur Respir J. 2012;40:1324–1343. 22743675 10.1183/09031936.00080312 PMC3786581

[15] Pellegrino R, Viegi G, Brusasco V, et al. Interpretative strategies for lung function tests. Eur Respir J. 2005;26:948–968. 16264058 10.1183/09031936.05.00035205

[16] Janaudis-Ferreira T, Hill K, Goldstein RS, Wadell K, Brooks D. Relationship and responsiveness of three upper-limb tests in patients with chronic obstructive pulmonary disease. Physiother Can. 2013;65:40–43. 24381380 10.3138/ptc.2011-49 PMC3563375

[17] Lima VP, Almeida FD, Janaudis-Ferreira T, Carmona B, Ribeiro-Samora GA, Velloso M. Reference values for the six-minute pegboard and ring test in healthy adults in Brazil. J Bras Pneumol. 2018;44:190–194. 30043884 10.1590/S1806-37562017000000388 PMC6188694

[18] Skumlien S, Hagelund T, Bjortuft O, Ryg MS. A field test of functional status as performance of activities of daily living in COPD patients. Respir Med. 2006;100:316–323. 15941658 10.1016/j.rmed.2005.04.022

[19] Van Remoortel H, Raste Y, Louvaris Z, et al. Validity of six activity monitors in chronic obstructive pulmonary disease: a comparison with indirect calorimetry. PLoS One. 2012;7:e39198. 22745715 10.1371/journal.pone.0039198 PMC3380044

[20] Santos-Lozano A, Hernandez-Vicente A, Perez-Isaac R, et al. Is the SenseWear armband accurate enough to quantify and estimate energy expenditure in healthy adults? Ann Transl Med. 2017;5:97. 28361062 10.21037/atm.2017.02.31 PMC5360603

[21] Corrêa KS, Karloh M, Martins LQ, Santos K, Mayer AF. Can the glittre ADL test differentiate the functional capacity of COPD patients from that of healthy subjects? *Braz J* Phys Ther. 2011;15:467–473. 10.1590/s1413-35552011005000034 22094546

[22] Hayran M, Hayran M. Sağlık araştırmaları için temel istatistik [Basic statistics for health research]. Omega Yayınları; 2011.

[23] de Camargo AA, Boldorini JC, Holland AE, et al. Determinants of peripheral muscle strength and activity in daily life in people with bronchiectasis. Phys Ther. 2018;98:153–161. 29237078 10.1093/ptj/pzx123

[24] Lima VP, Brooks D, Konidis S, et al. Normative values for the unsupported upper limb exercise test and 6-minute pegboard and ring test in healthy Canadian adults. Physiother Can. 2020;72:330–336. 35110804 10.3138/ptc-2019-0021 PMC8781495

[25] Cakmak A, Sonbahar-Ulu H, Inal-Ince D, et al. Impaired arm exercise capacity and increased energy expenditure during activities of daily living in patients with bronchiectasis compared to controls. Eur Respir J. 2019;54:PA1171.

[26] Levison H, Cherniack RM. Ventilatory cost of exercise in chronic obstructive pulmonary disease. J Appl Physiol. 1968;25:21–27. 5661150 10.1152/jappl.1968.25.1.21

[27] Wasnik MP, Shinde N. Comparison of physiological response to glittre ADL test and 6 minute walk test in patients with COPD: observational study. *Int J Health Sci Res.* 2019;9:125–130.

[28] Ozalp O, Inal-Ince D, Calik E, et al. Extrapulmonary features of bronchiectasis: muscle function, exercise capacity, fatigue, and health status. Multidiscip Respir Med. 2012;7:3. 22958327 10.1186/2049-6958-7-3 PMC3415114

[29] Karloh M, Karsten M, Pissaia FV, de Araujo CL, Mayer AF. Physiological responses to the glittre-ADL test in patients with chronic obstructive pulmonary disease. J Rehabil Med. 2014;46:88–94. 24104462 10.2340/16501977-1217

[30] Karloh M, Araujo CL, Gulart AA, Reis CM, Steidle LJ, Mayer AF. The glittre-ADL test reflects functional performance measured by physical activities of daily living in patients with chronic obstructive pulmonary disease. Braz J Phys Ther. 2016;20:223–230. 27437713 10.1590/bjpt-rbf.2014.0155 PMC4946838

[31] Gulart AA, Munari AB, Klein SR, Gavenda SG, Sagrillo LM, Mayer AF. Performance in the glittre-ADL test is associated with the pulmonary function of patients with chronic obstructive pulmonary disease. COPD. 2021;18:637–642. 34865582 10.1080/15412555.2021.2008339

[32] Ozsoy I, Ozcan Kahraman B, Ozsoy G, et al. Determinants of the 6-minute pegboard and ring test as an unsupported upper-extremity exercise capacity measure in older adults with chronic obstructive pulmonary disease. Eur Geriatr Med. 2018;9:863–870. 34674474 10.1007/s41999-018-0111-x

